# Brain metastasis-related microRNAs in patients with advanced breast cancer

**DOI:** 10.1371/journal.pone.0221538

**Published:** 2019-10-11

**Authors:** Jun Sato, Akihiko Shimomura, Junpei Kawauchi, Juntaro Matsuzaki, Yusuke Yamamoto, Satoko Takizawa, Hiromi Sakamoto, Makoto Ohno, Yoshitaka Narita, Takahiro Ochiya, Kenji Tamura

**Affiliations:** 1 Department of Breast and Medical Oncology, National Cancer Center Hospital, Tokyo, Japan; 2 Toray Industries, Inc., Kanagawa, Japan; 3 Division of Molecular and Cellular Medicine, National Cancer Center Research Institute, Tokyo, Japan; 4 Department of Biobank and Tissue Resources, Fundamental Innovative Oncology Core, National Cancer Center Research Institute, Tokyo, Japan; 5 Department of Neurosurgery and Neuro-Oncology, National Cancer Center Hospital, Tokyo, Japan; 6 Department of Molecular and Cellular Medicine, Institute of Medical Science, Tokyo Medical University, Tokyo, Japan; Universitatsklinikum Hamburg-Eppendorf, GERMANY

## Abstract

Brain metastasis is a major distant metastasis occurring in patients with advanced breast cancer, and is associated with poor prognosis. MicroRNAs (miRNAs) have a strong influence on various oncological functions and have been reported as potential biomarkers for detecting distant metastasis. Specific biomarkers and unique miRNAs for brain metastasis have yet to be reported. The aim of this study was to identify novel miRNAs in serum, to assist in the diagnosis of brain metastasis in patients with advanced breast cancer. We retrospectively analyzed the medical records of patients with breast cancer and collected clinical data. In addition, we evaluated serum miRNA profiles in patients with breast cancer, with and without brain metastasis, using high-sensitivity microarrays. All patients underwent computed tomography or magnetic resonance imaging brain imaging tests. A total of 51 serum samples from patients with breast cancer and brain metastasis, stored in the National Cancer Center Biobank, were used, and 28 serum samples were obtained from controls without brain metastasis. Two miRNAs, miR-4428 and miR-4480, could significantly distinguish patients with brain metastasis, with area under the receiver operating characteristic curve (AUC) values of 0.779 and 0.781, respectively, while a combination of miR-4428 and progesterone receptor had an AUC value of 0.884. No significant correlations were identified between the expression levels of these two miRNAs in serum and clinical data. We conclude that serum miR-4428 and miR-4480 may be useful as biomarkers for predicting brain metastasis in patients with breast cancer.

## Introduction

Breast cancer is one of the leading causes of death and the most common malignancy among women in developed countries. Up to 30% of patients with advanced breast cancer develop brain metastasis (BM), a type of distant metastasis associated with poor prognosis [1,2].

Recent advances in the development of drug therapies have improved the prognosis for patients with advanced breast cancer. However, those with BM still have poor outcomes. Previous studies have reported a median survival time for patients with breast cancer with BM, who received standard treatments for BM, of 6–15 months from BM detection [2,3]. The median survival time for patients with BM who received only steroids was approximately 2 months [4], while the introduction of radiation therapy for BM improved survival to approximately 6–7 months [5]. Further, chemotherapy is effective, to some extent, for patients with breast cancer and BM. Bachelot et al. reported that the overall survival among patients with advanced breast cancer with BM treated with platinum-based chemotherapy was 17.0 months (3.2–24.9 months) [6]. These previous reports suggest that an intensive treatment strategy, including radiation and chemotherapy, is critically important for patients with BM. In addition, Lin et al. indicated that poor performance status (Eastern Cooperative Oncology Group; ECOG) at the time of BM diagnosis was significantly associated with inferior outcomes following chemotherapy [7]; however, it is not uncommon for patients with BM not to receive radiation or chemotherapy for BM, because of the devastating effects on the general health status of the patient caused by BM. Considering the cost and time required to perform imaging tests, simplified detection of BM would provide clinical benefits for prevention and treatment.

Circulating microRNAs (miRNAs) are a class of small non-coding RNAs in serum/plasma that are 21–25 nucleotides long and can strongly influence various oncological functions, including both tumor suppression and cancer development [8]. Many circulating miRNAs have been reported as potential biomarkers for evaluation of breast cancer characteristics, including detection of the primary site in early stage disease or distant metastasis [9,10,11–18,19] [20–22].

In the context of distant metastasis, expression of several specific miRNAs in breast cancer specimens or cell lines is associated with metastatic localization [23,24]. The analysis of tissue samples from patients with breast cancer and BM revealed that levels of miR509, miR20b, miR181-c, and miR130b-3p were significantly correlated with BM [25–28]. Moreover, there is evidence that some of these miRNAs have roles in the mechanisms underlying BM; however, specific biomarkers and miRNAs unique to BM have yet to be reported.

Circulating miRNAs are detectable in serum samples and are potentially practical biomarkers [29], and have been proposed as promising biomarkers in patients with advanced breast cancer [21]. The aim of this study was to identify novel miRNAs in serum useful for diagnosing BM in patients with advanced breast cancer using a circulating miRNA array-based approach.

## Materials and methods

### Clinical samples

The medical records of patients with advanced breast cancer who were admitted or referred to the National Cancer Center Hospital (NCCH), Japan, between 2009 and 2016, were analyzed retrospectively. Patients with advanced breast cancer with the following characteristics were included: (1) pathologically diagnosed with breast cancer; (2) brain imaging test, including enhanced computed tomography (CT) scan and/or magnetic resonance imaging (MRI) conducted, regardless of the neurological symptoms; (3) treatment for breast cancer was performed in the NCCH; (4) serum samples were obtained within 1 month around the time when the imaging test was performed; and (5) patients with BM had not received any treatment (chemotherapy, radiation therapy, or surgery) before collection of serum samples. Serum samples from patients with breast cancer who provided consent for inclusion in the Biobank were stored at -20°C in the NCCH.

Clinical data, including sex, age, histology, hormone status, and human epidermal growth factor receptor (HER)-2, at the time of serum sample collection were investigated. Hormone status, estrogen receptor (ER), and progesterone receptor (PgR) were scored from 0 to 3 (0, no tumor cells stained; 1, < 1% stained; 2, > 1 ≤ 10% stained; 3, ≥ 10% tumor cells stained) by immunohistochemistry. In the present study, hormone status positivity was defined as a score of 3. Positivity for HER-2 was defined as follows: immunohistochemistry, 3+ (uniform intense membrane staining of > 30% of invasive tumor cells); fluorescence in situ hybridization, amplified ratio of HER-2 to CEP17 of > 2.2; or average HER-2 gene copy number, > 6 signals/nucleus for those test systems without an internal probe control, according to the American Society of Clinical Oncology (ASCO)/College of American Pathologists (CAP) guideline 2007 [30].

### Microarray analysis of miRNA expression

Total miRNA was extracted from 300 μl aliquots of serum samples using the 3D-Gene^®^ RNA extraction reagent from a liquid sample kit (Toray Industries, Inc., Kanagawa, Japan). Comprehensive miRNA expression analysis was performed using a 3D-Gene^®^ microRNA Labeling kit and a 3D-Gene^®^ Human microRNA Oligo Chip (Toray Industries, Inc.), designed to detect 2540 miRNA sequences registered in miRBase releases 20 and 21 (http://www.mirbase.org/). MiRNA was defined as present if the corresponding microarray signal was more than the signal (mean + 2 × standard deviations) of the negative controls, of which the top and bottom 5%, ranked by signal intensity, were considered noise generated by the experimental system and were removed. When the signal value was negative, or undetected, after background subtraction, the value was replaced by 0.1 on a base 2 logarithm scale. Three pre-selected internal control miRNAs (miR-149-3p, miR-2861, and miR-4463) were used to normalize signals across the different microarrays tested. The results were subjected to probability analysis using receiver operating characteristic curves. All microarray data of this study are in agreement with the Minimum Information About a Microarray Experiment (MIAME) guidelines and are publicly available through the GEO database (GSE134108, http://www.ncbi.nlm.nih.gov/projects/geo/).

### Statistical analysis

Samples were divided into two groups: those from patients with BM and controls (without BM). MiRNAs were compared between the two clinical groups using a two-sided Student’s t-test with Bonferroni correction. P-values < 0.01 were considered statistically significant. To identify robust biomarkers, only miRNAs that had a signal value of 2^6^ in more than 50% of analyses for each clinical group were selected for further analysis.

MiRNAs associated with BM were analyzed using multivariable logistic regression analysis models, by adjusting for possible risk factors for BM (age, hormone status, HER-2 status, resection surgery of primary site, and distant metastatic lesions). Variables included in the models were based on existing knowledge of breast cancer characteristics. Multicollinear analysis and determination of a linear model with univariable analysis (Pearson’s product-moment correlation coefficient) were performed. This was followed by assessment of the fit of the model and its performance characteristics. To evaluate the predictive performance of the model, a Hosmer-Lemeshow test was used. All statistical analyses were performed using SPSS version 19 (SPSS, Chicago, IL, USA).

### Ethical statement

The present study was approved by the NCCH Institutional Review Board. Written informed consent was obtained from all participants.

## Results

### Characteristics of patients and samples

**Fig 1:**
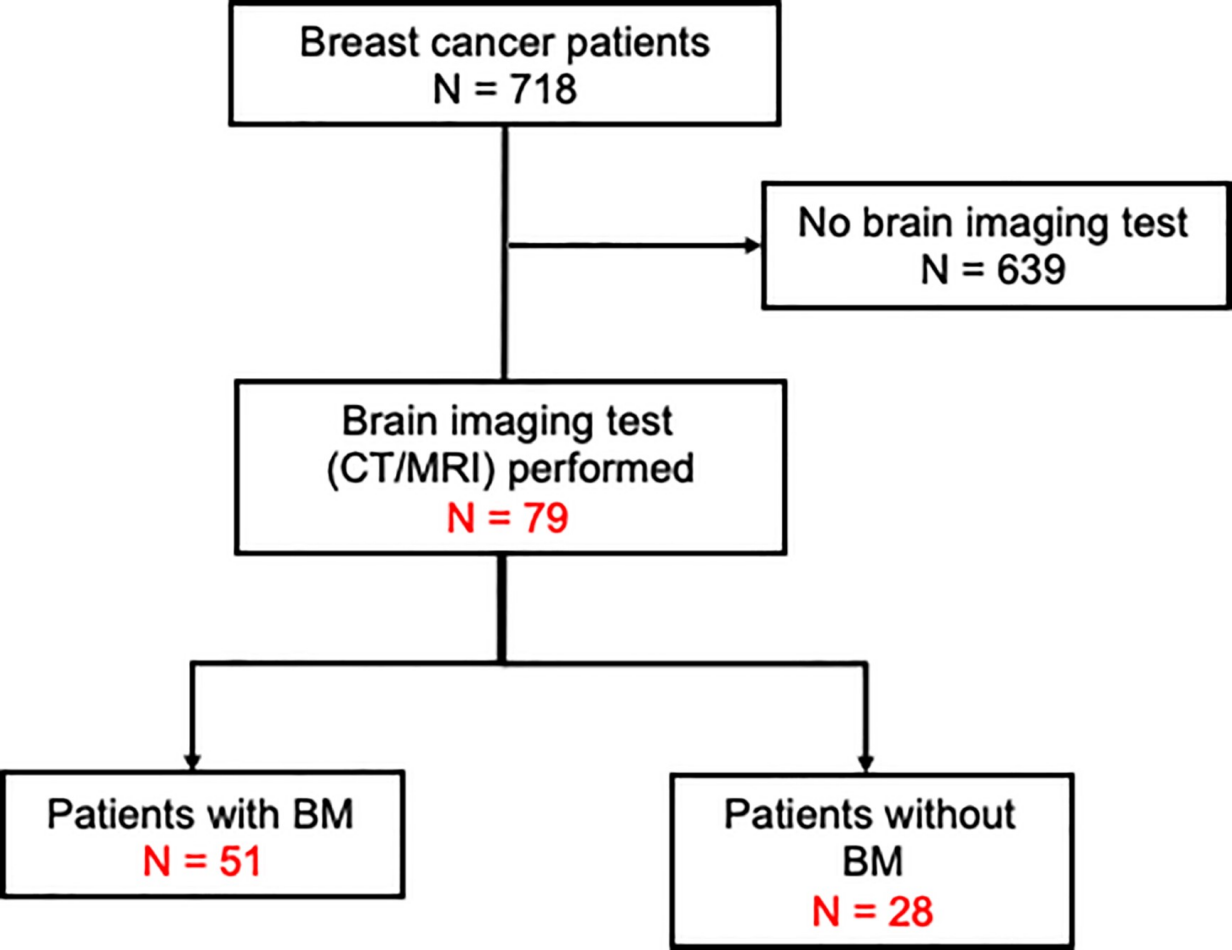
Consort diagram of the present study. CT/MRI, computed tomography/magnetic resonance imaging; BM, brain metastasis.

A total of 51 serum samples from patients with breast cancer and BM stored in the National Cancer Center Biobank, and 28 serum samples from patients with breast cancer without BM (controls), were included in the study. The consort diagram for the present study is presented in Fig 1. Patients who were registered with the present study had advanced (Stage 4) clinical stage disease or recurrent status after surgical resection. There were no male patients. The characteristics of the patients with breast cancer with or without BM are presented in Table 1. BM was confirmed by enhanced CT scan, simple MRI, or enhanced MRI. At the time of diagnosis of BM, none of the patients had received treatment for BM.

**Table 1:** Characteristics of patients with breast cancer, with or without brain metastasis.

	With BM	Without BM	P
Total number of patients	51	28	
Age (years), median (range)	55 (27–82)	49 (35–85)	0.294
Pathology			0.383
IDC	39	24	
ILC	2	0	
Apocrine	1	0	
Mucinous	1	0	
Others	7	4	
NA	1	0	
Hormone-sensitive status			
PgR			0.001
Positive	11	17	
Negative	37	11	
NA	3	0	
ER			0.005
Positive	15	18	
Negative	33	10	
NA	3	0	
Ki-67 (%); median (range)	41.8 (11–84)	43.3 (6–92)	0.024
HER-2 status (IHC)			0.092
Positive	15	4	
Negative	32	24	
NA	4	0	
Number of brain metastases		NA	
Single	17		
Multiple (≧2)	34		
Mean size (max) of brain metastases (mm)	30.0	NA	
Primary tumor (not resected)	12	10	0.253
Distant metastases			
Lung	26	8	0.055
Liver	15	15	0.035
Lymph node	26	18	0.260
Bone	17	15	0.082
Skin/soft tissue	5	4	0.555
Pleural	2	4	0.099

BM, brain metastasis; ER, estrogen receptor; HER-2, human epidermal growth factor receptor 2; IDC, invasive ductal carcinoma; ILC, invasive lobular carcinoma; IHC, immunohistochemistry; NA, not applicable; PgR, progesterone receptor.

### Comparison of patients with and without BM

**Fig 2:**
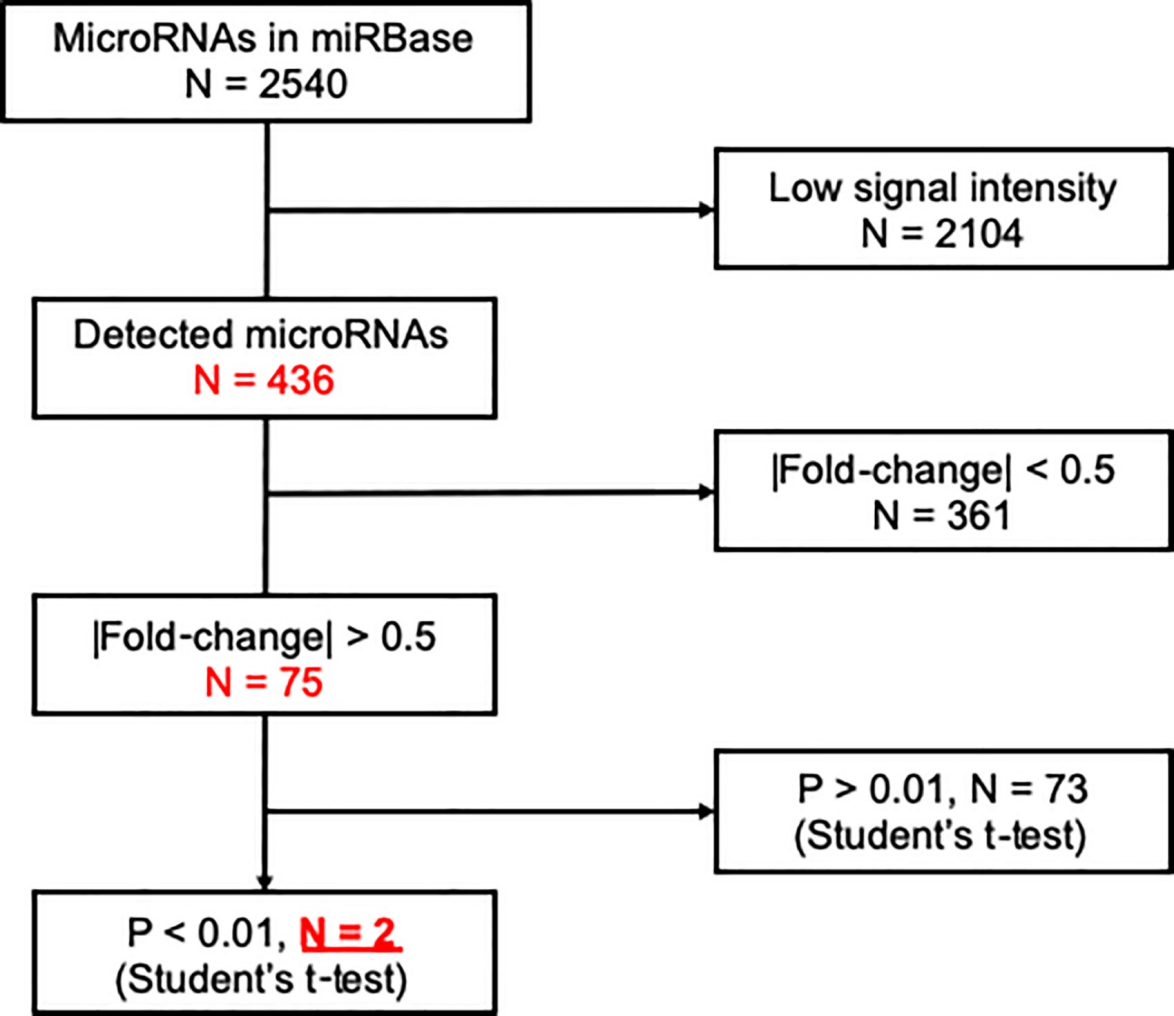
Flow diagram for detection of microRNAs.

**Fig 3:**
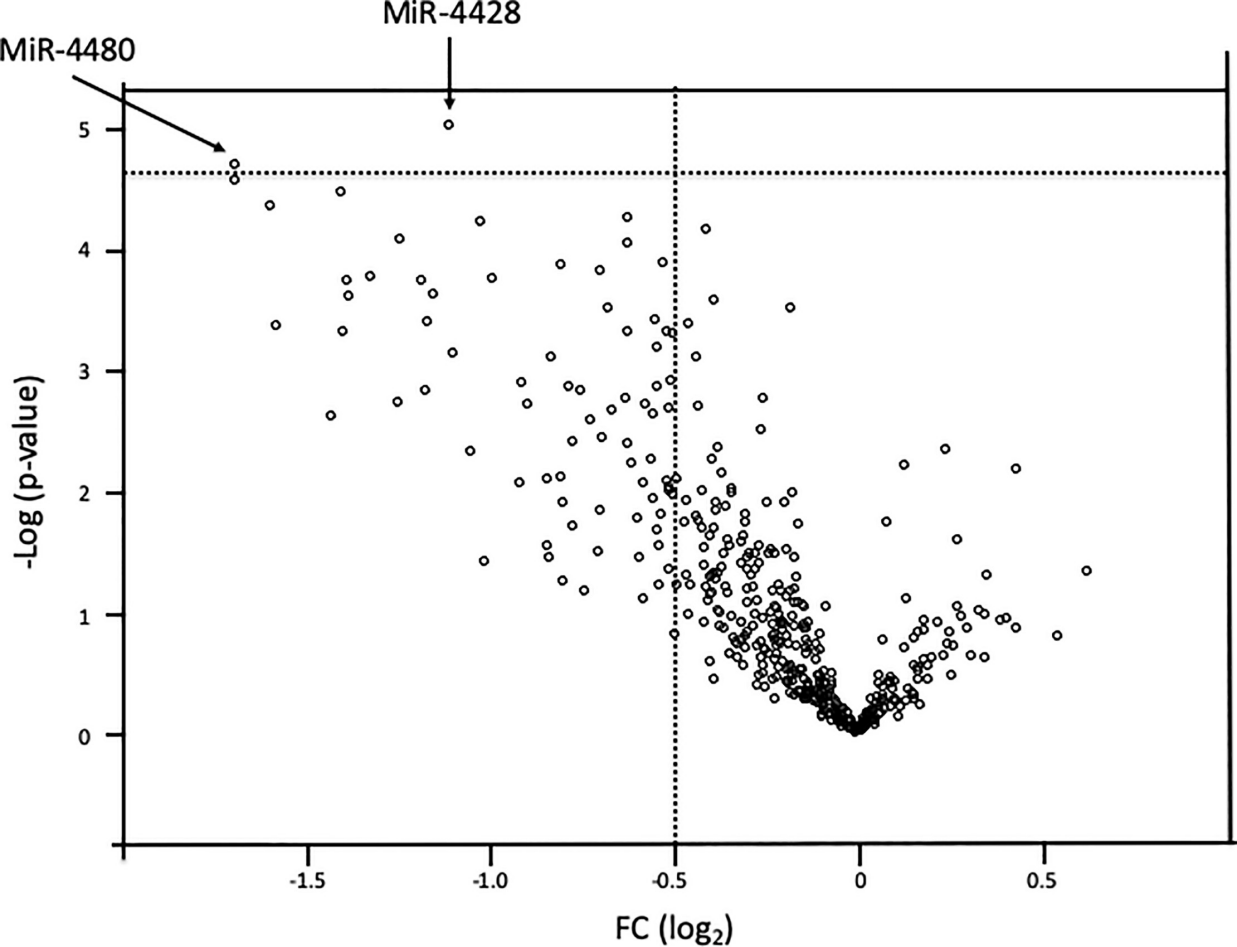
Volcano plots of microRNAs. FC, fold-change. Vertical dashed line shows the border of >0.5 |foled change| Horizontal dashed line shows the border of >0.01 p-value.

To identify promising miRNA biomarkers, we excluded miRNAs with low signal intensity (< 2^6^) from the miRNA microarray data (Fig 2). Next, we conducted two-sided Student’s t-tests, with Bonferroni correction, to compare data from patients with and without BM; the results from 436 detected miRNAs are plotted as a volcano plot (Fig 3). According to our selection criteria (> 0.5 |fold-change| and p < 0.01), two miRNAs, miR-4428 and miR-4480, were selected as candidate biomarkers for diagnosis of BM in patients with breast cancer (Fig 2).

**Fig 4:**
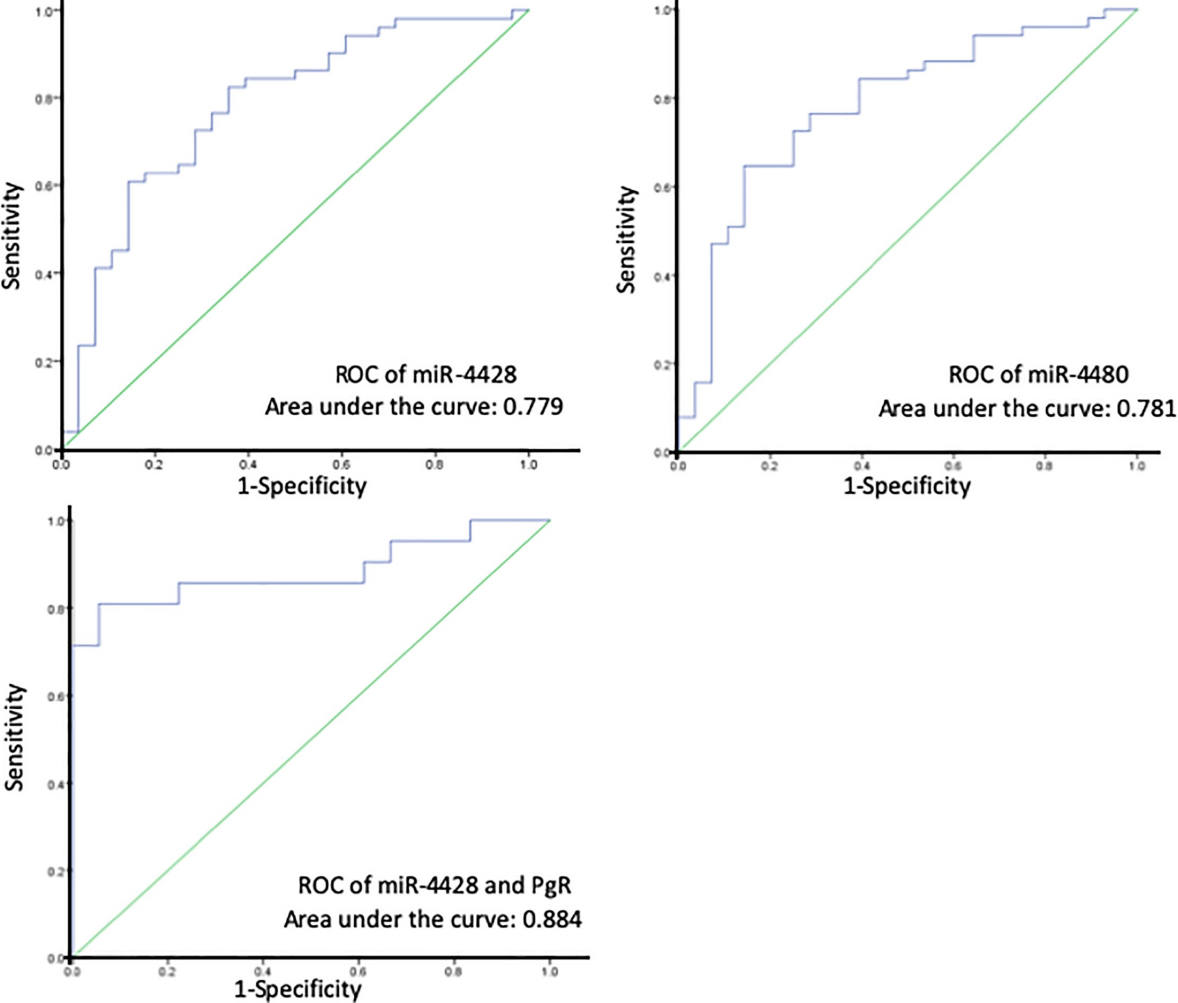
Receiver operating characteristic curve (ROC) analysis of the diagnostic index using miR-4428, miR-4480, and a combination of miR-4428 and progesterone receptor (PgR).

Receiver operating characteristic curves for the test cohort are presented in Fig 4. Area under the curve (AUC) values for miR-4428 and miR-4480 were 0.779 and 0.781, respectively, while a combination of miR-4428 and PgR had an AUC of 0.884. Specificity and AUC values were calculated for the discrimination of patients with BM from non-BM controls. miR-4428 and miR-4480 showed sensitivity of 82.4% and 76.5% and specificity of 64.3% and 71.4%, respectively.

### Multivariable analysis for candidate miRNAs

To further investigate the two identified miRNAs (miR-4428 and miR-4480) as potential biomarkers for BM, we performed multivariable logistic regression model analysis. First, we estimated the linearity of each dependent variable, using Pearson’s product-moment correlation coefficient. No strong linearity was identified among these variables. Multiple logistic regression analysis, adjusted for clinical characteristics and tumor properties, suggested a regression model including miR-4480 and PgR (Table 2). Pearson’s chi-square test for multivariable models indicated statistical significance (p < 0.001), as did tests for each dependent variable (p < 0.001). In addition, analysis using the Hosmer-Lemeshow statistic suggested borderline significance (p = 0.143). The predictive value of the model was 84.6%, and no predicted value exceeded ± 3 standard deviations of the measured value. These results suggested that miR-4480 is a strong candidate biomarker for prediction of BM and that PgR status may influence the expression of miR-4480 and BM.

**Table 2:** Results of multivariable analysis.

	Univariable analysis		Multivariable analysis	
	Odds ratio (95% CI)	P	Odds ratio (95% CI)	P
Age	1.009 (0.971–1.048)	0.654		
ER	0.253 (0.094–0.676)	0.006		
PgR	0.192 (0.070–0.530)	0.001	0.111 (0.020–0.623)	0.013
HER-2	2.812 (0.828–9.558)	0.098		
Ki-67 (%)	0.981 (0.961–1.001)	0.062		
Primary site (not resected)	0.554 (0.202–1.518)	0.251		
Distant metastases				
Lung	2.600 (0.969–6.974)	0.058		
Liver	0.361 (0.139–0.940)	0.037		
Lymph node	0.578 (0.224–1.491)	0.257		
Bone	0.433 (0.169–1.113)	0.082		
Skin/soft tissue	0.652 (0.160–2.656)	0.551		
Pleural	0.245 (0.042–1.432)	0.118		
miR-4428	2.847 (1.660–4.883)	< 0.001	2.752 (1.249–5.300)	0.010
miR-4480	1.888 (1.344–2.652)	< 0.001		

CI, confidence interval; ER, estrogen receptor; HER-2, human epidermal growth factor receptor 2; NA, not applicable; PgR, progesterone receptor.

## Discussion

This is the first report to reveal the prognostic function of serum miRNAs for brain metastases in breast cancer patients. Several miRNAs are specifically expressed in breast cancer BM tissues; however, there are no reports of detection of BM-associated miRNAs in serum samples, with the exception of miR-4428 and miR-4480, reported in this study.

Recently, a number of new cancer-related miRNAs have been identified, the majority of which have never been examined for their applicability to detection of BM in patients with breast cancer. Wolf et al. measured the expression levels of miRNAs by qRT-PCR in BM samples from patients originally diagnosed with unknown primary carcinoma, and reported that specific miRNAs of primary tumor origin were expressed in metastatic samples [31]. Moreover, a number of miRNAs have been indicated to have functions in patients with various types of cancer [32,33]. No functional association of miR-4428 and miR-4480 with clinical data has previously been reported in patients with breast cancer and distant metastasis. One report suggested an association between miR-4480 and the pathogenesis of age-related macular degeneration; however, the underlying mechanism remains unknown [34]. There is a lack of reports suggesting roles for, or detailing distribution of, miR-4428.

MiRNAs have many important functions in both the promotion and suppression of cancer pathogenesis [8]. Although the mechanisms underlying their involvement in distant metastasis, including BM, have yet to be defined, some miRNAs have been associated with specific functions in breast cancer. According to a study of breast cancer cell lines, miR-7 expression is involved in the migration and invasion of tumor cells, through regulation of the expression of the *KLF4* gene[23]. Associations of miRNAs with BM and destruction of the blood brain barrier (BBB) have been demonstrated in more than one report. MiR-181c was found to lead abnormal distribution of actin, thereby inducing functional changes in the BBB, associated with cancer-derived extracellular vesicles in cell lines derived from breast cancer BM [27]. In a different pathway, miR-1258 suppresses breast cancer metastasis to the brain, and alterations in this miRNA were indicated to influence the mechanism of BM and BBB function [24,35]. The roles of the miRNAs identified in the present study in the mechanisms underlying BM remain unclear, and further functional analyses are required to address this question.

Several specific miRNAs expressed in distant metastasis have been reported in breast cancer cells. A previous report showed that miR-106b-5p is expressed in pulmonary metastasis tissue samples and may be a predictive marker for lung metastasis [22]. Further, the combination of upregulation of miR-30b and downregulation of miR-125b is associated with lymph node metastasis [36]. Similarly, there is evidence that miR-135 and miR-203 can impact tumor growth in the bone microenvironment, through influencing RUNX2 protein levels [37,38]. MiR-218 effects the mechanism underlying bone metastasis through activation of the WNT signaling pathway in breast cancer [39]. While conducting multivariable analysis in the present study, we did not detect any relationship between miR-4428 (which was associated with BM) and other distant sites. In the few studies that have investigated the role of serum miRNAs in the mechanism of invasion and metastasis, distant lesions were investigated.

Several miRNAs useful for detecting early stage breast cancer were not identified in this study [21,40]. According to a study conducted by Silvia et.al., miR-484 is a potential biomarker for early stage breast cancer; although this study reported novel findings, based on analysis of serum miRNAs, the small number of samples (39 patients with breast cancer and 10 healthy volunteers) was a limitation of the investigation. Shimomura et al. reported that a combination of five miRNAs (miR-1246, miR-1307-3p, miR-4634, miR-6861-5p, and miR-6875-5p) has a sensitivity of 97.3% and specificity of 82.9% for detecting early stage breast cancer by comparing 1280 samples from patients with breast cancer, 2836 from non-cancer controls, 451 from other types of cancer, and 63 from non-breast benign diseases [21]. As it is difficult to obtain samples from intracranial tumors in a general clinical setting, and as early detection of BM influences the accessibility to less-invasive radiation treatment, a stereotactic radiosurgery etc., investigation of miR-4428 and miR-4480 as specific serum biomarkers for BM in patients with breast cancer is potentially valuable for earlier diagnosis and accurate monitoring for preventing deterioration of general condition.

In the current study, univariable analysis, in the course of multivariable analysis, suggested that ER, PgR, and the presence of liver metastasis may be inversely related to BM. Few studies have indicated significant relationships between hormone status and BM [41]. According to one report, among 14,599 breast cancer patients with BM, the ER positive rate was 37% (41% negative and the others unknown), the PgR positive rate was 36% (34% negative), the HER-2 positive rate was 35% (41% negative), and the triple negative rate was 27% (18% triple positive) [2]. MiR-106b was correlated with poor prognosis in triple negative breast cancer [42]. Based on the results of these studies, the association among BM, hormone status, and miRNAs remains unclear.

The present study has certain limitations, including the small sample size in each group and the fact that tissue sample analyses were not conducted. In the clinical setting, brain imaging tests are not performed routinely, due to the low frequency of BM, which (along with the difficulty in obtaining samples from brain tumor tissue) may explain the small sample size. In addition, these factors make it difficult to investigate the origin of serum miRNAs. It will be necessary to evaluate the expression levels of miRNAs compared with those in primary tumor tissue, metastatic site tissue, and serum. A further, prospective investigation, including functional analysis, should be conducted.

## Conclusion

This is the first study to report the serum microRNAs as possible biomarker of BM in breast cancer. Serum miR-4480 and PgR negative are useful biomarkers for predicting BM in patients with breast cancer. Serum miR-4428 could be a biomarker and further investigation might be warranted.
